# Prevalence and Characteristics of Smokers Interested in Internet-Based Smoking Cessation Interventions: Cross-sectional Findings From a National Household Survey

**DOI:** 10.2196/jmir.2342

**Published:** 2013-03-18

**Authors:** Jamie Brown, Susan Michie, Tobias Raupach, Robert West

**Affiliations:** ^1^Cancer Research UK Health Behaviour Research CentreDepartment of Public Health and EpidemiologyUniversity College LondonLondonUnited Kingdom; ^2^Department of Clinical, Educational and Health PsychologyUniversity College LondonLondonUnited Kingdom; ^3^National Centre for Smoking Cessation and TrainingLondonUnited Kingdom; ^4^Department of Cardiology and PneumologyUniversity Medical Centre GottingenGottingenGermany

**Keywords:** smoking cessation intervention, Internet-based, website, prevalence, characteristic

## Abstract

**Background:**

An accurate and up-to-date estimate of the potential reach of Internet-based smoking cessation interventions (ISCIs) would improve calculations of impact while an understanding of the characteristics of potential users would facilitate the design of interventions.

**Objective:**

This study reports the prevalence and the sociodemographic, smoking, and Internet-use characteristics of smokers interested in using ISCIs in a nationally representative sample.

**Methods:**

Data were collected using cross-sectional household surveys of representative samples of adults in England. Interest in trying an Internet site or “app” that was proven to help with stopping smoking was assessed in 1128 adult smokers in addition to sociodemographic characteristics, dependence, motivation to quit, previous attempts to quit smoking, Internet and handheld computer access, and recent types of information searched online.

**Results:**

Of a representative sample of current smokers, 46.6% (95% CI 43.5%-49.6%) were interested in using an Internet-based smoking cessation intervention. In contrast, only 0.3% (95% CI 0%-0.7%) of smokers reported having used such an intervention to support their most recent quit attempt within the past year. After adjusting for all other background characteristics, interested smokers were younger (OR=0.98, 95% CI 0.97-0.99), reported stronger urges (OR=1.29, 95% CI 1.10-1.51), were more motivated to quit within 3 months (OR=2.16, 95% CI 1.54-3.02), and were more likely to have made a quit attempt in the past year (OR=1.76, 95% CI 1.30-2.37), access the Internet at least weekly (OR=2.17, 95% CI 1.40-3.36), have handheld computer access (OR=1.65, 95% CI 1.22-2.24), and have used the Internet to search for online smoking cessation information or support in past 3 months (OR=2.82, 95% CI 1.20-6.62). There was no association with social grade.

**Conclusions:**

Almost half of all smokers in England are interested in using online smoking cessation interventions, yet fewer than 1% have used them to support a quit attempt in the past year. Interest is not associated with social grade but is associated with being younger, more highly motivated, more cigarette dependent, having attempted to quit recently, having regular Internet and handheld computer access, and having recently searched for online smoking cessation information and support.

## Introduction

The World Health Organization recently attributed 12% of all global deaths among adults aged 30 years and over to tobacco [1]. Almost all these deaths could be avoided if smokers quit before their mid-30s [2]. Yet, in many countries such as the United Kingdom, less than a quarter of smokers quit by this age despite the majority wanting and trying to stop [3,4]. The most effective interventions involve face-to-face behavioral support combined with medication such as nicotine replacement therapy or varenicline [5-7]. However, even in England, where there is a universally available behavioral support program, the vast majority of smokers do not use face-to-face support and almost half attempt to stop unaided [8]. The Internet could be an ideal medium for helping those people who do not wish, or are unable, to engage in face-to-face behavioral support [9,10].

Behavioral support delivered via the Internet has the advantage that it is extremely cost-effective, and some patients prefer the increased convenience and confidentiality and reduced stigma [11,12], while others who are less able to access face-to-face support because of either mobility or geographical barriers may also find it useful. The benefits of Internet support over other low-cost and convenient alternatives to face-to-face support, such as written materials, include the capacity for interactivity and tailoring. Additionally, researchers and practitioners should be attracted by the capability to disseminate evidence-based support faithfully and flexibly update content to reflect new information as it emerges [13].

There is extensive evidence that the Internet can be an effective delivery mode for the behavioral support of a variety of health issues [11,14], and the United Kingdom has issued guidance to use particular programs in routine clinical care (eg, Beating the Blues for mild and moderate depression and FearFighter for phobia, panic, and anxiety) [15]. More importantly, there is also specific evidence from three separate systematic reviews that Internet-based smoking cessation interventions (ISCIs) can help smokers to quit compared with brief written materials or no intervention [16-18]. Current evidence is somewhat limited by the heterogeneity of effect across different interventions, insufficient reporting of content [19-21], and the paucity of data relating to long-term abstinence with biochemical verification of smoking status, yet research work is underway that may be able to address these limitations (eg, StopAdvisor [22,23]). In the context of this modest evidence of efficacy, together with the unique advantages of ISCIs, such as low cost, it is important to identify the prevalence of smokers who would be interested in using such support.

An accurate estimate of the likely reach of ISCIs is necessary for calculations of impact [24]. Previous estimates of potential reach have often been based on either national figures for Internet access or reported interest among nonrepresentative samples [25,26]. One study that did assess a representative sample of smokers estimated that 40% were interested in using an ISCI [27]. However, the study was conducted between 2006-07, and Internet access and usage patterns are relatively fast-moving phenomena [28]. For example, in Britain the percentage of households that have at least one method of using the Internet while at home increased from 58% in 2003 to 70% in 2009 and again to 77% in 2011 [28,29], while the use of wireless Internet hotspots doubled in just 12 months to 4.9 million users in 2011 [29].

Understanding the characteristics of smokers interested in using ISCIs may help the development of new interventions, or modification of existing ones, in several regards including tailoring dimensions, choice of content and features, navigational architecture, and language style and complexity. Similarly, designers would be interested in these associated characteristics for the purpose of dissemination, particularly online advertising, which can often be targeted to reach, or at least focus on, only certain demographic groups. Previous studies have characterized individuals who search for cessation information [30] and who use Internet interventions [31-35], smokers on their use of the Internet [36], and smokers who were either invited to, eligible for, or enrolled in cessation programs according to their subsequent use of the interventions [37-39]. While it is clearly essential to understand these profiles, particularly what determines use among those who are already interested, in order to improve the appeal of these cessation interventions it is also important to establish how interested smokers compare with those who are not in nationally representative samples. To our knowledge, only one other study has characterized a representative sample of smokers on the basis of their interest in ISCIs [27]. In that study, younger and more cigarette dependent smokers who had better Internet access were more likely to express an interest. However, there was no assessment of other important smoking characteristics such as current motivation to stop and past quit attempts, nor was there an assessment of recent online searching behavior.

This study addressed the following research questions:

1. How many smokers in a nationally representative sample are interested in using ISCIs?
2. What smoking, Internet use, and sociodemographic characteristics are associated with interest in the use of these interventions?

## Methods

### Study Design

The data were taken from the Smoking Toolkit Study [40], which is an ongoing series of cross-sectional household surveys in England designed to provide information about smoking prevalence and behavior. Each month a new sample of approximately 1800 adults aged 16 and over completes a face-to-face computer-assisted survey with a trained interviewer. By conducting a face-to-face rather than online survey, Internet access should not confound the results. Taylor Nelson Sofres-British Market Research Bureau collects the data as part of their monthly omnibus surveys on behalf of researchers at the Cancer Research UK’s Health Behaviour Research Centre, University College London, who conceived of the study and continue to manage it. The surveys use a form of random location sampling. England is split into 165,665 Output Areas, each comprising approximately 300 households. These Output Areas are stratified by A Classification Of Residential Neighbourhoods (ACORN) characteristics (an established geo-demographic analysis of the population provided by CACI International) and then randomly selected to be included in the lists of the interviewers. Interviewers travel to the selected areas and perform interviews with one participant per household until quotas based upon factors influencing the probability of being at home (working status, age, and gender) are fulfilled. Morning interviews are avoided to maximize participant availability. These survey methods have been previously described and have been shown to result in a baseline sample that is nationally representative in its sociodemographic composition and proportion of smokers [40]. Ethical approval was granted by the University College London ethics committee.

### Participants

We used data from respondents to the survey between February 2012 and April 2012 who reported smoking cigarettes (including hand-rolled) daily or occasionally at the time of the survey. A total of 5405 adults were surveyed; 1190 reported currently smoking cigarettes regularly of whom 1128 had complete data on all relevant variables.

### Measures

Current smokers were asked: “If there were an Internet site that was proven to help with stopping smoking, how likely is it that you would try it?” and also “If there were an application (“app”) for your handheld computer (like a “smartphone” [eg, an iPhone, Blackberry, or Android phone], palmtop, PDA, or tablet) that was proven to help with stopping smoking, how likely is it that you would try it?”. For the purposes of analysis, smokers’ responses on 4-point scales were dichotomized as either being “interested” in using an Internet-based smoking cessation intervention (ie, those responding “very likely” or “quite likely” to either question) or “not interested” (ie, those responding “very unlikely” or “quite unlikely” to both questions).

Additionally, current smokers were asked questions that assessed gender, age, and social grade (AB = higher and intermediate professional/managerial, C1 = supervisory, clerical, junior managerial/administrative/professional, C2 = skilled manual workers, D=semi-skilled and unskilled manual workers, E=on state benefit, unemployed, lowest grade workers), dependence (Heaviness of Smoking Index, HSI [41] and Strength of Urges [42]), motivation to quit (Motivation to Stop Scale [43]), previous attempts to quit smoking, access to the Internet and handheld computers, and recent types of information searched online, that is, “For which of the following activities did you use the Internet in the last 3 months for private use? Please indicate all that apply: (a) using services related to travel and accommodation, (b) reading or downloading online newsnewspapersnews magazines, (c) looking for a job or sending a job application, (d) seeking health related information or support other than stopping smoking (eg, injury, disease, nutrition, improving health, etc), (e) seeking stop-smoking related information or support, (f) looking for information about education, training or courses, (g) doing an online course (in any subject), (h) consulting the Internet with the purpose of learning, (i) finding information about goods or services” [44]. Responses to Items (a) to (c) and (f) to (i) were aggregated to calculate a variable identifying use of the Internet for information other than health related (Item d) or smoking cessation (Item e).

### Analysis

Data were analyzed using PASW 18.0.0. We used weighted data only to estimate the prevalence of interest in ISCIs among all smokers regardless of their Internet access. Data were weighted using the rim (marginal) weighting technique to match English census data on age, sex, and socioeconomic group. To assess smoking, Internet use, and sociodemographic characteristics associated with interest in the use of ISCIs, we conducted a series of simple and multiple logistic regressions. Alpha was set at *P*<.05.

## Results

Approximately 70% of current smokers had accessed the Internet in the past week while a significant majority also had access to a handheld computer (see Table 1). A minority of users had searched for either smoking or health information support, and more than half had searched for at least one of a variety of “other” types of online information. The sociodemographic and smoking characteristics were typical of a representative sample of smokers [4,40], and by way of comparison, the characteristics of the 4451 current smokers included in the Smoking Toolkit Study for the 12 months before the current study (ie, January 2011 to January 2012) are presented in Table 1.

A total of 42.6% (95% CI 39.6%-45.7%) of current smokers were interested in using Internet sites for smoking cessation, 23.9% (95% CI 21.3%-26.5%) were interested in apps, and 46.6% (95% CI 43.5%-49.6%) were interested in ISCIs (either sites or apps). In contrast, only 0.3% (95% CI 0%-0.7%) of smokers reported having used such an intervention to support their most recent quit attempt within the past year.

Table 2 shows the smoking, Internet use, and sociodemographic characteristics of smokers by their interest in the use of ISCIs. There was evidence that interested smokers were younger, more cigarette dependent (measured by both HSI and Strength of Urges), more motivated to quit within 3 months, more likely to have made a quit attempt in the past year, accessed the Internet at least weekly, had handheld computer access, had used the Internet to search for online smoking cessation information or support in past 3 months, and had used it to search for a variety of “other” online information. After adjusting for all other background characteristics, associations remained between interest and age, cigarette dependence (measured by Strength of Urges), motivation to quit, past year quit attempt, weekly Internet access, handheld computer access, and recent searching for online smoking cessation information. Last, this pattern of results was unchanged during sensitivity analyses in which the associations between interest and the various characteristics were re-assessed separately when smokers were classified according to whether or not they had expressed interest in (1) an Internet site, or (2) an app (data not shown).

**Table 1:** Characteristics of current smokers.

	Current sample of Smoking Toolkit Study: Feb. ‘12 to Apr. ‘12 (n=1128)	Population of Smoking Toolkit Study: Jan. ‘11 to Jan. ‘12 (n=4451)
Mean (SD) age	41.7 (16.7)	43.0 (16.9)
% (N) women	47.6 (537)	49.7 (2210)
% (N) social grade C2DE	72.4 (817)	69.9 (3112)
Mean (SD) heaviness of smoking index	2.0 (1.5)	2.1 (1.5)
Mean (SD) strength of urges score	2.1 (1.0)	2.1 (1.1)
% motivated to quit within 3 months	20.3 (229)	22.9 (1020)
% quit attempt in past year	30.9 (348)	30.5 (1358)
% using face-to-face behavioral support in most recent quit attempt within past year	1.3 (15)	1.1 (49)
% access Internet at least weekly	70.1 (791)	—
% handheld computer access	41.6 (469)	—
% seeking online cessation information or support in past 3 months	3.0 (34)	—
% seeking online health (not smoking) information or support in past 3 months	9.5 (107)	—
% seeking “other” online information or support in past 3 months	63.5 (716)	—

**Table 2:** Factors associated with interest in Internet-based smoking cessation interventions.

	Interested (n=488)	Not interested (n=640)	OR (95% CI)	Adj. OR (95% CI)
Mean (SD) age	36.6 (13.5)	45.6 (17.9)	0.97 (0.96-0.97)^a^	0.98 (0.97-0.99)^a^
% (N) women	47.3 (231)	47.8 (306)	0.98 (0.77-1.24)	1.06 (0.81-1.38)
% (N) social grade C2DE	69.5 (339)	74.7 (478)	0.77 (0.59-1.00)	0.98 (0.73-1.32)
Mean (SD) heaviness of smoking index	2.1 (1.5)	1.9 (1.5)	1.10 (1.02-1.19)^a^	1.10 (1.00-1.22)
Mean (SD) strength of urges score	2.3 (1.0)	2.0 (1.0)	1.33 (1.18-1.50)^a^	1.29 (1.10-1.51)^a^
% motivated to quit within 3 months	29.3 (143)	13.4 (86)	2.67 (1.98-3.60)^a^	2.16 (1.54-3.02)^a^
% quit attempt in past year	41.0 (200)	23.1 (148)	2.31 (1.78-2.99)^a^	1.76 (1.30-2.37)^a^
% using face-to-face behavioral support in most recent quit attempt within past year	1.8 (9)	0.9 (6)	1.99 (0.70-5.62)	0.96 (0.28-3.27)
% access Internet at least weekly	85.9 (419)	58.1 (372)	4.37 (3.24-5.90)^a^	2.17 (1.40-3.36)^a^
% handheld computer access	56.4 (275)	30.3 (194)	2.97 (2.32-3.80)^a^	1.65 (1.22-2.24)^a^
% seeking online cessation information or support in past 3 months	5.3 (26)	1.2 (8)	4.45 (1.99-9.91)^a^	2.82 (1.20-6.62)^a^
% seeking online health (not smoking) information or support in past 3 months	11.3 (55)	8.1 (52)	1.44 (0.96-2.14)	0.82 (0.53-1.29)
% seeking “other” online information or support in past 3 months	77.7 (379)	52.7 (337)	3.13 (2.40-4.07)^a^	1.43 (0.97-2.10)

^a^ *P*<.05.

## Discussion

Almost half of all current smokers were interested in using an Internet-based smoking cessation intervention, however less than 1% had used one to support their most recent quit attempt in the past year. After adjustment for all background characteristics, smokers who were younger, more dependent, highly motivated to quit, had attempted to quit recently, accessed the Internet regularly, had handheld computer access, and had recently searched for online smoking cessation information or support were all more likely to be interested in using online stop smoking support.

The diffusion of the Internet since its inception over 40 years ago has been phenomenal—recently, the Internet reached a billion users worldwide [28]. In Britain, Internet access has continued to increase with 77% of households connected in 2011 as compared to 58% in 2003 [28,29]. Although smokers tend to have less access than nonsmokers [27], there remains a majority of smokers that would be possible to reach via the Internet—in this study over 70% had used the Internet in the past week—and this number is only likely to increase [28]. As a consequence, ISCIs are often cited as offering a valuable opportunity to deliver low-cost behavioral support to large numbers of smokers [16,21,25,45]. Importantly, this study now adds an up-to-date estimate of the proportion of smokers interested in using these interventions, which provides some indication of their maximum potential reach and should allow more accurate calculations of the likely impact of particular interventions [24]. For example, from the RE-AIM perspective, a public health impact score can be represented as a multiplicative combination of reach, efficacy, adoption, implementation, and maintenance. The accuracy of this calculation for particular ISCIs may be improved by the provision in the current paper of an up-to-date estimate of the denominator necessary to calculate the reach, which is defined as the proportion of the possible target population that participate in a particular intervention.

The current estimate of 47% of smokers who are interested in ISCIs is higher than the 40% previously estimated from a representative sample of smokers [27]. However, that study was conducted in 2006-07, and it is reasonable to assume that interest may have increased as a consequence of improving Internet access [28]. Additionally, interest in ISCIs was operationalized as an expression of interest in either an Internet site or app—the number reporting interest only in Internet sites was 43%. Last, the first study was conducted in Canada, and it is likely there are cultural differences in interest as compared to England.

The finding that interested smokers were likely to be younger and use the Internet more regularly is consistent with previous research [27]. This association with age is particularly important as treatment-seeking smokers tend to be older than those who do not seek treatment [46], and therefore online interventions may be particularly suitable for targeting younger smokers who may otherwise attempt to quit unaided. In contrast, the association between interest and dependence, motivation, and past year quit attempts is characteristic of smokers who are more likely to seek treatment [46-48]. It is important that any future assessments of the real-world effectiveness of ISCIs take these associations into account [48].

The association between interest and recent searching for online smoking cessation information or support is intuitive. However, it is also indicative of the potential demand for these interventions in that smokers do not appear to be deterred nor satisfied by what they are currently finding. The latter point is also suggested by the contrast between the 3% of smokers who recently searched for online smoking cessation information or support as compared with the 0.3% who used online support during a quit attempt in the past year.

The “digital divide” that characterizes Internet use in the wider population is also true of smokers: smokers who use the Internet tend to be more affluent and educated than those who do not [36]. Therefore, the interest of smokers regardless of social grade in the current study is an unexpected finding and suggests the Internet may yet offer a means of equitable treatment delivery, which is particularly important as smokers from more deprived socioeconomic groups typically want, and try, to stop as much as other smokers but find it more difficult [49].

A potential concern with ISCIs is that it might prevent smokers from using face-to-face support, which is currently the most effective delivery mode for behavioral support [5-7]. While relatively few smokers had recently used face-to-face support in the current study, the lack of an association between use of this support and interest tentatively suggests the concern is unwarranted. Instead, it is likely that Internet support would appeal to a different subset of smokers who place more value on convenience and confidentiality, or find other types of support difficult to access [11]. Additionally, in other health areas where Internet support has become routine, the two have emerged as complementary, for example in several areas of mental health online cognitive behavioral therapy is frequently recommended while a patient waits for an appointment or to help with the more automated aspects of the support [11].

Taken together, the pattern of associations provided in the current study present a comprehensive characterization of smokers interested in using ISCIs. This understanding is important as access cannot primarily account for the difference between interest in and use of these interventions; for example, in the current study the majority of smokers had used the Internet in the past week. It is hoped that these associations will inform the development or modification of interventions with regards to targeted dissemination, tailoring dimensions, choice of content and features, navigational architecture, and language style and complexity, and in turn help to actualize the potential of the Internet for delivering smoking cessation interventions [50,51].

An indirect point of interest is the finding that in simple logistic regressions, interest was associated with both measures of dependence: Strength of Urges and HSI. However, the association with only Strength of Urges in multiple logistic regression is consistent with previous research showing that a single-rating measure of urges may be a more useful measure of dependence than those based on consumption [42].

One possible limitation is that an expression of interest during a survey is clearly quite different from actually visiting a program to support a quit attempt and subsequently using the program regularly. However, the purpose of the current study was primarily to provide an estimate of the maximum possible reach if the support was more widely available and promoted. One of the important findings is the huge potential in terms of the difference between the proportion who are interested and the percentage currently using. Additionally, establishing the characteristics associated with interest is arguably critical to realizing this potential and improving uptake by facilitating effective targeting, both in terms of advertising and intervention content, and complements research into understanding the determinants of use among the small proportion currently using, eg, [37-39]. Future research should aim to derive the specific relationship between interest and subsequent uptake of ISCIs following targeted design and promotion. Another limitation is that the questions used to derive interest in ISCIs were framed positively (eg, “If there were an Internet site or ‘app’ that was proven to help with stopping smoking, how likely is it that you would try it?”). However, even if interest is overestimated, it is improbable that it would account for the extent of the discrepancy between interest in and use of ISCIs, which is highlighted by the current study. Another potential limitation may have been to operationalize interest in ISCIs as an expression of interest in either an Internet site or an app. While both require the Internet and a computer for delivery, there are also clear differences, including that only sites require regular Internet access and only apps require ongoing access to a handheld computer. Indeed, fewer smokers were interested in apps as compared to Internet sites; however, similar proportions of smokers who had regular Internet access and those with ongoing access to handheld computers were interested in sites and apps respectively. More importantly, in sensitivity analyses in which smokers were characterized separately according to interest in either sites or apps, the pattern of results was unchanged. Together these results suggest that the current operationalization of ISCIs for the purposes of estimating and characterizing interest is suitable; however, experimental research is needed to establish whether there are important differences in efficacy according to delivery mode of either Internet site or app. A final limitation is potential error or bias in the measurement of certain smoking characteristics. For example, smokers often forget failed quit attempts, particularly if they only lasted a short time or occurred long ago [52]. However, it is unlikely that forgetting would differ as a function of interest in ISCIs, and quit attempts—as with all other variables of interest in the present study—were assessed with a validated measure.

In conclusion, almost half the smokers in England are interested in using online smoking cessation interventions, and yet only a small proportion of smokers currently use these interventions to support quit attempts. Clearly, ISCIs represent an excellent opportunity to deliver low-cost behavioral support to a large number of smokers, which is currently not being realized. Moreover, as interest is expressed regardless of social grade, the Internet may also offer a means of delivering this support equitably. Last, designers of Internet-based interventions should be aware that potential users are likely to be younger, more cigarette dependent, highly motivated, have attempted to quit recently, have regular Internet and handheld computer access, and have recently searched for smoking cessation information and support.

## Abbreviations

ACORN: A Classification of Residential Neighbourhoods
HSI: heaviness of smoking index
ISCI: Internet-based smoking cessation interventions
RE-AIM: reach, efficacy, adoption, implementation, and maintenance
